# Effects of Mixing Techniques and Material Compositions on the Compressive Strength and Thermal Conductivity of Ultra-Lightweight Foam Concrete

**DOI:** 10.3390/ma17112640

**Published:** 2024-05-30

**Authors:** Tongyu Xu, Harald Garrecht

**Affiliations:** Institute of Construction Materials, University of Stuttgart, 70569 Stuttgart, Germany; tongyu.xu@iwb.uni-stuttgart.com

**Keywords:** foamed concrete, mineralized foam, thermal conductivity, rheological properties

## Abstract

The research focuses on ultra-lightweight foam concrete with a dry density below 200 kg/m^3^, primarily used as insulation material. Factors that may affect material properties are categorized into mixing techniques and material composition, and experimental investigations were conducted on the impact of these factors on the rheological properties of cement slurry, density at different time intervals, compressive strength, and thermal conductivity of foam concrete samples. The experimental results indicate the influence of mixing speed and mixing duration on the instrument during the cement slurry production and mixing process with foam. Additionally, variations in foam concrete sample properties are observed due to the water-to-cement ratio, foam content, and foam density in the selected material compositions. By analyzing the material density at different time intervals, the relationship between the ambient air trapped during the mixing process and the viscosity of the material can be indirectly observed. This analysis can also reveal the correlation between the unplanned air content and the properties of the material.

## 1. Introduction

### 1.1. Motivation

The energy crisis is a perennial and extensively studied topic, with indicators such as energy consumption and greenhouse gas emissions driving research in various fields. In recent years, due to changes in real-world conditions, energy conservation and emissions reduction have transcended mere verbal discourse. They have become pressing issues, propelled by multifaceted demands and new policies.

The United Nations, the International Energy Agency (IEA), and the Global Alliance for Buildings and Construction (GlobalABC) annually release global statistics on energy consumption and greenhouse gas emissions. According to the IEA’s 2018 report, it explicitly states that the building sector accounted for 36% of global final energy use and nearly 40% of energy-related carbon dioxide emissions in 2017 [1]. With policy momentum and research advancements, the GlobalABC 2022 report highlights that in 2021, the building sector accounted for 34% of global energy consumption and 37% of the total global carbon dioxide emissions. Considering that the measurements are in exajoules (EJ) for energy and gigatons (Gt) for carbon dioxide, each percentage represents a significant quantity. However, simultaneously, it also highlights the immense potential within the building sector for energy conservation and emissions reduction [2].

To meet the continually evolving requirements for carbon neutrality, there are essentially two methods available. First, increase the proportion of clean energy sources such as solar, wind, and tidal energy in the overall energy consumption or advance the research and development of new energy sources, such as bioenergy, that have not yet met usage conditions, to unleash their potential. Second, reduce the waste of existing energy resources and make full use of the available energy. In the 2022 IEA report, it is highlighted that the EU region reduced carbon emissions by 2.5% in 2022, equivalent to 70 Mt. The significant decrease in emissions in the building sector is attributed to the relatively mild winter conditions [3]. This is sufficient evidence that, through more meticulous design and the use of appropriate materials, energy consumption in the construction sector can be effectively reduced.

### 1.2. Introduction to Mineralized Foam

In the field of construction, various types of insulation materials are employed to reduce the thermal conductivity of building structures and enhance energy efficiency. Among the most commonly used materials are Polyurethane Foam (PU), Expanded Polystyrene (EPS), Extruded Polystyrene (XPS), and others. Each of these materials has its advantages, but numerous studies have underscored their issues. The primary concern revolves around the fact that their raw materials are largely derived from fossil products. Additionally, popular residential and building insulation materials like stonewool and glasswool also demand significant energy consumption in their production processes [4]. These conventional insulation materials pose serious environmental impacts throughout their entire production phases. Furthermore, upon reaching the end of their service life, they continue to contribute harm as they are often non-recyclable, taking several decades to centuries to naturally decompose [5]. This presents a pressing challenge in terms of sustainable and eco-friendly building practices.

Concrete, as the most widely used material in the construction industry, has always been a subject of considerable research. The issue of recycling concrete, in particular, has been a hot topic, with numerous successful cases of using recycled concrete [6]. Among these, foam concrete, as a type of lightweight concrete, has attracted significant attention. Its preparation process involves introducing air bubbles into the concrete at the stage before the material hardens. Under the condition that the bubble structure in the material remains stable, it eventually hardens and forms a porous structure through the hydration reaction of the binder, such as cement [7]. The higher air content in foamed concrete not only reduces overall density but, crucially, maintains a stable bubble structure, ensuring excellent thermal insulation properties [8]. Compared to traditional synthetic materials, this method not only addresses the issue of non-recyclability but also, due to the introduction of a significant volume of air, ensures the production of a considerable volume of foamed concrete with minimal raw materials. This dual advantage makes foamed concrete an ideal choice, especially in situations requiring lightweight structures and effective thermal insulation [9]. However, at the same time, it has been shown that the influences on the properties of foam concrete, in addition to the different raw materials in the recipes, are to a large extent limited by the uncertainties in the production process. Consequently, in situations where extreme demands are placed on the thermal insulation properties of the material, traditional polymer materials are often preferred [10]. However, a large number of studies on foam concrete have also shown that the thermal conductivity of foam concrete is proportional to the density of the material [11,12,13]. In the existing studies on foam concrete, the density design interval of the samples tends to be in the range of 300 kg/m^3^ to 1800 kg/m^3^, below which the studies are, to a certain extent, vacant. This leads one to wonder whether foam concrete can achieve the same extreme thermal insulation properties as polymer materials in the lower density range.

The objective of this study is to conduct a comprehensive analysis of mineralized foam materials with a density below 200 kg/m^3^. Various factors influencing the performance of the final samples will be investigated to lay the groundwork for future improvements. The emphasis will be placed on exploring the characteristics and properties of mineralized foam materials.

## 2. Materials and Methods

The main properties analyzed in this study concern the rheological characteristics of the uncured material, primarily focusing on yield strength and viscosity. Additionally, the density of the cured material, its corresponding compressive strength, thermal conductivity, and the pore structure of the material were studied.

### 2.1. Materials

#### 2.1.1. Cement

Typically, Ordinary Portland Cement (OPC), as the traditional binder, is the preferred choice in most studies involving concrete materials; however, considering the high clinker content in OPC and the substantial energy consumption and significant CO_2_ emissions associated with producing this clinker [14,15], the study has selected Optimo 5R cement, which is a Portland composite cement product with a density of 3.05 kg/dm^3^ from Holcim, located in Rostock, Germany. Its material compositions are tabulated in Table 1. Because of the reduced cement clinker content, it is generally more environmentally friendly than OPC, while also taking the availability of materials into account.

#### 2.1.2. Foaming Agent

It belongs to an artificially synthesized surfactant, mainly composed of 2-butoxyethanol and sodium lauryl ether sulfate. During the foaming process using SBM-8, its concentration was set at 2.5 wt%.

### 2.2. Test Variables and Mix Proportions

In this study, experiments and analyses were conducted on two aspects of mineralized foam: material composition and mixing techniques. For instance, in terms of material composition, variables included the water–cement ratio, the density of foam used for mixing, and its proportion with cement paste. Concerning mixing techniques, control was exercised over the duration and speed of mixing during cement paste production, as well as the mixing duration and speed of the ELBA mixer when blending cement paste with foam. These variables are outlined in Table 2, while the proportions measured through experimental tests are listed in Table 3. In Table 3, the symbols rpm and min represent both the mixing speed and the mixing time of the relevant mixer, as well as the units of these two variables. Notably, the foam density can be regulated by adjusting the air and water intake of SBM-8, although achieving precision in these adjustments can be challenging. Therefore, only design density is tabulated in Table 2 and Table 3.

### 2.3. Measurement Devices

#### 2.3.1. Rheological Charateristics

For the analysis of rheological properties, the study utilized the device RN 4.1 from RHEOTEST Medingen GmbH, Ottendorf-Okrilla, Germany. Rheometers are broadly classified into various types, encompassing shear or rotational rheometers, extensional rheometers, acoustic rheometers, falling plates, and capillary/contraction flow [16,17].

The equipment belongs to the shear rheometer category, and for different materials, adaptation can be achieved by changing the device’s rotor, each with different recommended viscosity and rotational speed ranges. For measuring the yield strength and viscosity of the cement slurry used in this study, a cylindrical rotor conforming to DIN EN 53019-1 [18] was used. The experiments on the rheological properties of the cement paste were carried out with reference to the experimental procedure described in [19]. Before each experiment, a fixed amount of 35 mL of cement paste material was measured and filled into a tub, which was set in a temperature-regulating device. The rotor was first rotated at a constant speed of 50 rpm for a duration of 10 min to ensure that the temperature of the cement paste at the time of measurement was about 20 degrees Celsius and to minimize the potential influence of temperature on the material’s rheological properties. After the process of temperature regulation, the rotor speed is increased uniformly from 0 to 300 rpm over a period of 3 min.

The equipment measures the torque ”through controlled shear strain rates and integrated torque sensors, then generates real-time shear rate-stress plots, based on which the viscosity and yield strength of the material are derived using the Bingham fluid model.

#### 2.3.2. Compressive Strength

The compressive strength testing on the samples was conducted using a combined testing machine from TESTING Bluhm & Feuerherdt GmbH, Berlin, Germany.

Considering the density of the samples and the possible damage to the pore structure due to water pressure, the samples are not kept underwater but stored in an air environment with a temperature of approximately 20 °C and a relative humidity of 65%. The samples were tested for compressive strength at 7 and 28 days with reference to DIN EN 12390-3 [20]. However, considering that the compressive strength of the foam concrete is expected to be very low, the load rate was set at 0.1 MPa/s, which was inspired by [21].

#### 2.3.3. Thermal Conductivity

In this study, THB 100 equipment from Linseis Messgeraete GmbH, Selb, Germany, was utilized to ensure compliance of devices with [22,23,24] standards. The measurement principle of this equipment is referred to as the Transient Hot Bridge method, an adaptation derived from the Transient Hot Wire method [25,26,27] and classified under the time-domain thermoreflectance (TDTR) approach, which has been proven to be reliable in the use of a wide range of studies [28]. By inserting a sensor integrated with a heat source and a resistance thermometer into the sample, the sensor itself heats up and measures the temperature changes in the surrounding area, thereby deriving the material’s thermal conductivity and temperature conduction properties. For different materials, adaptability is achievable by replacing the sensors. The THB 6K sensor, utilized in this research, possesses a measurement range from 0.01 W/(mK) to 1 W/(mK), with an error margin within approximately ±3%.

### 2.4. Specimen Preparation

The specimens used in the study were prepared through a two-phase mixing process. In the first stage of mixing, cement paste was prepared by using the batch mixer CIM-30-E from Gertec Maschinen-und Anlagenbau GmbH, Sulzberg, Germany. At the end of the first stage of the mixing process, the required amount of cement paste was sent to the laboratory equipped with a rheometer for testing the rheological properties. At the same time, the remained cement paste, after deducting the amount for the rheological property tests and the losses left in the mixer, was mixed with the correspondingly designed quantity of preformed foam, which is made by the SBM-8 foaming machine from Gertec in the mixer CEM 60 S ELBA from AMMANN, Langenthal, Switzerland, to prepare the mineralized foam for subsequent experiments.

Samples for the compressive strength tests were manufactured according to the DIN EN 12390-3 [20] specifications, resulting in standard cubic samples measuring 150 × 150 × 150 mm^3^. These same specifications were used for the analysis of pore structures. Considering the material size’s impact on the precision of the THB 100 and the THB 6K sensor’s requirements, recommended sample sizes for the sensor were set at equal to or larger than 40 × 20 × 1 mm^3^. Taking into account the necessity of a flat surface for the sensor and considering the loss caused by cutting and polishing, samples for thermal conductivity measurements were produced as standard cubic shapes measuring 100 × 100 × 100 mm^3^.

For each set of different tests, three identical samples were prepared with the same specifications. All samples were demolded on the third day after mixing and subsequently stored in compliance with DIN EN 12390-2 [29] standards, awaiting further measurements. Aside from the 7-day and 28-day compressive strength tests, thermal conductivity and pore structure tests were conducted 30 days after sample fabrication. This time frame aimed to ensure the material properties and structure were predominantly formed, allowing the effects of hydration reactions to be essentially negligible. Moreover, the samples used for thermal conductivity testing were dried in an oven at 105 degrees Celsius for three days before testing. After removing the samples from the oven, they were wrapped in cling film to prevent moisture from infiltrating into the specimens, and the tests were performed after the internal temperature of the specimens had returned to room temperature.

## 3. Results

### 3.1. The Rheological Behavior of Cement Paste and Its Relationship to Water/Cement Ratio

Since most commercially available superplasticizers contain defoamer components, the use of superplasticizers to modulate the rheological properties of the cement paste has a high risk of resulting in the collapse of the foam structure in subsequent foam concretes made with the same batch of cement paste. Also, since the purpose of this study is, to some extent, to serve as a basis for future studies, neat cement paste with rheological properties influenced by adjusting the w/c ratio was chosen as the first option. And by adjusting the w/c ratio, a range between 0.4 and 1.3 was selected. The lower limit of 0.4 was chosen to theoretically ensure that there is enough water in the slurry to facilitate the hydration reaction of the cement. By doing so, it is expected that the possibility of the cement slurry drawing water from the foam for the hydration reaction can be reduced. If the cement draws water from the foam for hydration and there is not enough water in the mixture, theoretically, the thickness of the water film between the bubbles would decrease, potentially leading to further destruction of the bubble structure. In preliminary tests involving pure cement slurry, it was observed that when the w/c ratio was 0.9 or higher, water and cement slurry started to show separate layering within 15 min after the slurry was prepared. This 15-min timeframe generally exceeds the duration needed to mix the cement slurry with foam in each experimental group after the slurry has been prepared. To ensure that experimental data could be compared over a wide range and to observe the potential effects of layering in the cement slurry, the maximum w/c ratio was set at 1.3. 

**Figure 1:**
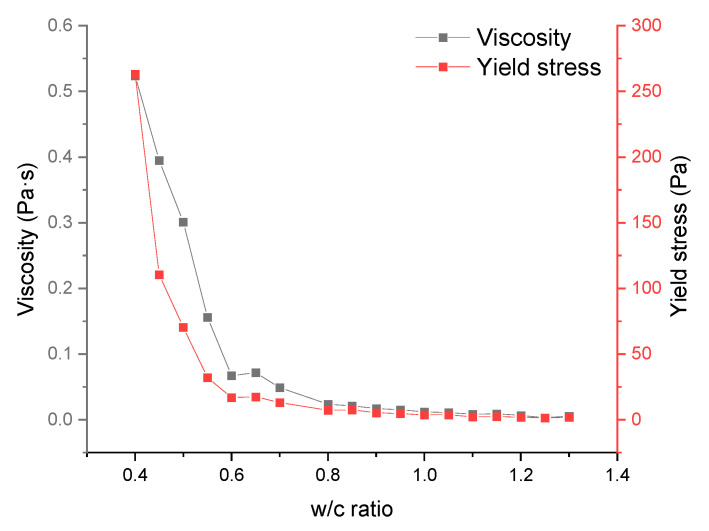
Experimental results of rheological properties.

It has been mentioned in [30] that the measured rheological properties of cement slurries are affected by a number of physical and chemical factors at the same time (e.g., the measuring apparatus, experimental procedure, temperature, the amount of material used in the test, water/cement ratio, size of the cement particles, composition of the slurry), therefore, although there are many studies covering the effect of water–cement ratio on the rheological properties of cement slurries [31], there are no available formulas or models to characterize the rheological properties of slurries incorporating all of the above variables. In this study, by controlling the used materials, experimental procedures, and other factors, the water–cement ratio was intentionally made to be the most significant variable. Through using the water–cement ratio as the independent variable and the viscosity as well as the yield strength of the cement paste as the corresponding strain variables, the experimental results were obtained as shown in Figure 1, both of which show an exponential decrease with the increase of the water–cement ratio, and similar exponential trends can also be seen within the data mentioned in [31,32]. Remarkably, this declining trend moderates within the range of 0.6 to 0.8 in the water/cement ratio. Higher ratios beyond this range show a minimally significant impact on the rheological properties of the cement paste.

### 3.2. Relationship between Material Properties and Water/Cement Ratio

Regarding the material’s various properties, the primary considerations are density, compressive strength, and thermal conductivity. The density of materials can be evaluated at different times. The measured density at the time of casting is denoted as fresh density *ρ_f_*; the density measured after 28 days under air curing conditions according to DIN EN 12390-2 is called *ρ_wet_*; the density measured after oven drying is termed *ρ_dry_* [33]. In addition to this, under the assumption that the air in the mixture comes only from the preformed foam added to the mixer during the second stage of mixing and that the content of air is kept constant during the mixing process, the desired fresh density of the mixture was obtained by calculating the following equation:

(1) $$\rho_{design,fresh}=\frac{m_{water}+m_{foam\;water}+m_{cement}}{V_{water}+V_{foam}+V_{cement}}$$

In this case, the density of water was taken as 1.0 g/cm^3^, the density of cement powder was taken as 3.05 g/cm^3^ given by the manufacturer, and the density of the foam was taken as the design value of 45 g/L (the preset value of the foaming machine was kept constant for each experiment, and the density was measured after the production of the foam in order to ensure that the density was within the tolerance of ±5 g/L). Since this design density points to the casting process, the volume loss of the concrete due to hydration reactions should be negligible here [34,35].

**Figure 2:**
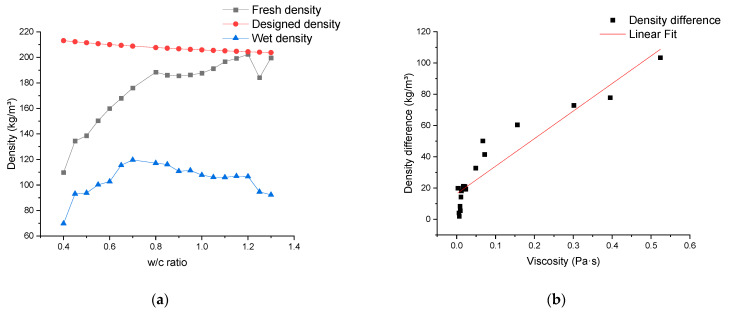

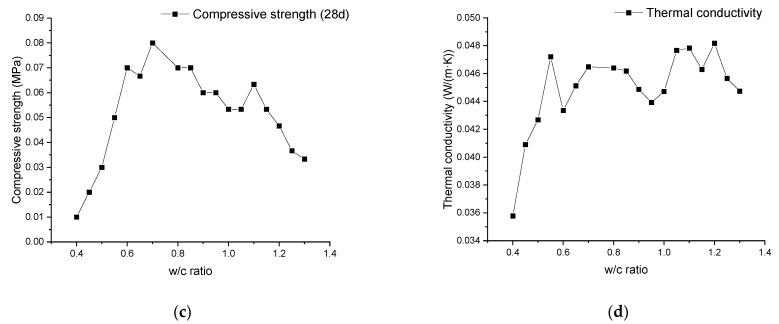
The experimental results depict the variation in the properties of mineralized foam concerning changes in the water/cement ratio. (**a**) Density; (**b**) density difference; (**c**) compressive strength; (**d**) thermal conductivity.

In Figure 2a, the designed density, fresh density, and wet density after 28 days of air drying are depicted. The designed density exhibits a linear relationship with the water/cement ratio with a slight downward trend. The fresh density first presents a significant increase within the water/cement ratio range of 0.4 to 0.8, after which the upward trend becomes slow. At the water–cement ratio of 1.15, the measured value of fresh density exceeds the design density for the first time. The wet density of the material exhibits an ascending trend within the range of water–cement ratios from approximately 0.4 to 0.7. It reaches its peak at a water–cement ratio of 0.7 and subsequently shows a stable descent. In addition, in Figure 2a, the variational trend of the difference between the designed density and the fresh density can be observed. This difference is termed the density difference ∆ρ, and in Figure 2b, it is correlated with the viscosity of the cement paste. The relationship between the two can be approximated by a linear equation, with R^2^ = 0.875. For the observed difference between the fresh density and the design density, there are the following speculations: During the material preparation process, the bubble structure of the material is disrupted during the mixing process, reducing the air content and causing the air to “escape” into the surrounding environment, leading to a decrease in the overall volume of the material and an increase in density. Conversely, without considering the mass loss caused by the evaporation of moisture in the material, air from the surrounding environment is drawn into the material, causing an increase in the overall volume of the material and a decrease in density. In the mixing process, theoretically, both of these scenarios should coexist. 

Connecting these speculations with the results presented in Figure 2b, the conclusion drawn is that the rheological properties of the cement slurry significantly influence the “exchange” between internal and external gases during the material mixing process. When the viscosity of the cement slurry is high, gases from the environment are drawn into the material and can be approximated as being trapped in the material. Conversely, in the process of mixing with a high water–cement ratio slurry, gases from the environment can escape after being drawn into the material. This allows for a better prediction of the fresh density concerning the design density. Similar evidence will be mentioned later in the experimental results on mixing time and the analysis of the material’s pore structure. 

With the inference regarding the impact of cement slurry viscosity/yield strength on the interaction of gases inside and outside the material, the changing trend of material wet density can be explained. During the process of increasing the water–cement ratio from 0.4 to 0.7, the trend of viscosity/yield strength with the water–cement ratio is significant. Their decrease leads to a reduction in the gas content captured from the surrounding environment, resulting in an increase in the wet density of the material. However, after the water–cement ratio exceeds 0.7, the trend of viscosity/yield strength with the water–cement ratio becomes less pronounced, reducing their impact on the gas content inside the material. At this point, the increase in the water–cement ratio leads to a rise in the water content within the material, and the influence of the water content on the wet density becomes predominant. As the water content increases, the amount of moisture that can be lost during the same preservation process also rises, causing a decreasing trend in the wet density of the material with the increasing water–cement ratio. However, it is noteworthy that, in comparison to the impact on fresh density, the variation in wet density of the material remains within a relatively narrow range.

In Figure 2c, the trend of compressive strength variation with water/cement ratio is depicted. With an increase in the water/cement ratio, the 28-day compressive strength of the mineralized foam exhibits an initial significant rise, reaching its peak at a water/cement ratio of 0.7, followed by a decline. This trend aligns with the variation in wet density depicted in Figure 2a, and it also corroborates the conclusion in [36,37,38] that the strength of foam concrete material is primarily influenced by its density.

The thermal conductivity performance with respect to the water–cement ratio is illustrated in Figure 2d. It can be observed that the thermal conductivity of the samples shows a significant increase in the range of the water–cement ratio from 0.4 to 0.6, followed by a fluctuating trend within a certain range. Considering that the thermal conductivity of foam concrete is influenced not only by the air content within the material [19,39] but also to a large extent by the pore structure [40,41], the following inference can be drawn: within the range where the water–cement ratio is below 0.6, the variation in air content inside the samples is apparent. In this process, the dominant effect of air content on thermal conductivity results in an increasing trend with the rising water–cement ratio. After surpassing a water–cement ratio of 0.6, as the impact of the water–cement ratio on air content diminishes, the thermal conductivity is mainly influenced by the material’s pore structure, displaying a fluctuating pattern. This conclusion can be further supported by subsequent experiments analyzing the pore structure.

Furthermore, with regard to the previously mentioned layered cement slurry observed in a high w/c ratio, there was no significant effect on any of the results. This may be because in the process of mixing the cement paste with the foam, the originally layered cement paste was once again stirred well, and part of the free water would eventually be dispersed in the water film in the foam structure, but the amount of this part of the free water was not enough to significantly change, for example, the thickness of the water film of the foam or to affect the performance of the mineralized foam. 

### 3.3. Relationship between Material Properties and Foam Density

**Figure 3:**
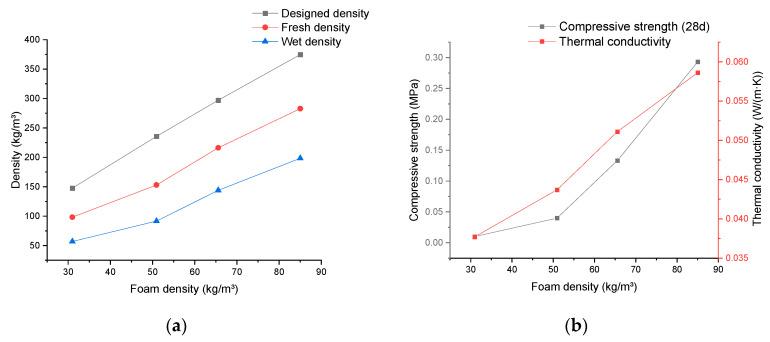
The experimental results depict the variation in the properties of mineralized foam concerning changes in the foam density. (**a**) Density; (**b**) thermal conductivity and compressive strength.

The corresponding results obtained by using foam density as the independent variable are depicted in Figure 3. In Figure 3a, the designed density, fresh density, and wet density for mineralized foam exhibit a linear upward trend with an increase in the selected foam density. These values show a nearly similar slope within the chosen density range.

Figure 3b demonstrates a similar ascending trend observed in both the thermal conductivity and compressive strength of the mineralized foam. The thermal conductivity change tends to approximate linearity, while the compressive strength shows exponential growth. 

It is worth noting that when the density of the foam used for mixing was the focus of the study, the mixing proportion of the cement paste used was kept consistent, with a water/cement ratio of 0.6, as shown in Table 3. This same proportion ensures that the rheological properties of the cement paste fluctuate only within a stable range, which allows the influence of the rheological properties of the paste on the properties of the foamed concrete to be controlled. From this, combined with the density difference ∆ρ between the designed densities $\rho_{design}\;$ and fresh densities $\rho_{f}$ of the foam concretes in Figure 3a between different foam densities, it can be indirectly concluded that the density of the foam used in the mixing of the slurry, or, more directly, the rheological properties of the fresh foam concrete have less influence on the amount of ambient air introduced into the material and also retained in the material during the mixing process.

### 3.4. Relationship between Material Properties and Foam Content

**Figure 4:**
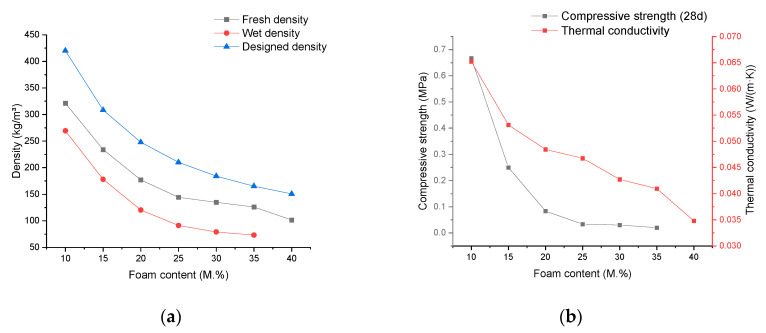
The experimental results depict the variation in the properties of mineralized foam concerning changes in the ratio of foam mass to the total mass of cement paste. (**a**) Density; (**b**) thermal conductivity and compressive strength.

The obtained results, considering the ratio of foam mass to the total mass of cement paste as the independent variable, are illustrated in Figure 4. In Figure 4a, there is a consistent decreasing trend in the designed density, fresh density, and wet density of mineralized foam with an increase in foam content. It is also worth noting here that the density difference between the design density and the fresh density decreases as the foam content of the material increases, but the amount of this change is not only quite small, but the density difference essentially ceases to change when the foam content exceeds 30% of the mass of the cement paste. This again supports the observation made in Section 3.3 that the introduction and retention of ambient air are minimally affected by the rheological properties of fresh foam concrete. Similarly, by comparing the difference between the fresh and wet densities of the foam concrete in Figure 3a as well as in Figure 4a, it can be observed that the density difference between the fresh and wet densities of the foam concrete remains roughly the same, whether one varies the content of the foam in the composition of the material or the water content of the foam itself. This leads to another inference: that the amount of water from the foam in the material composition has almost no impact on the quality of water lost through natural evaporation during the preservation process.

In Figure 4b, the variation of the material’s thermal conductivity and compressive strength with foam content is illustrated. Here, the thermal conductivity shows an almost linear relationship with foam content. In subsequent experiments observing the pores, it was found that at different foam contents, the material’s pore structure exhibited a similar state. From this, the inference is drawn that the linear relationship between thermal conductivity and foam content here is more influenced by the air content in the material, with the impact of the pore structure being secondary. A similar conclusion can be drawn regarding the limited influence of foam content changes on the transformation of the material’s pore structure. The compressive strength, on the other hand, shows a significant exponential decay trend with increasing foam content, and the downward trend of compressive strength almost stabilizes after exceeding 25% foam content. In [42], a series of experiments were also conducted using foam content as an independent variable to investigate the compressive strength of foam concrete. The results it presents show that as the foam content increases, the compressive strength continuously decreases, and the rate of decrease becomes faster. This differs from the trend observed in the current results with varying foam content. However, considering that it used materials containing fly ash and sand and that the density range of its samples was much higher than that of the current experiment, determining specific influencing factors becomes challenging.

### 3.5. Relationship between Material Properties and Mixing Technologies for Production of Cement Pastes

**Figure 5:**
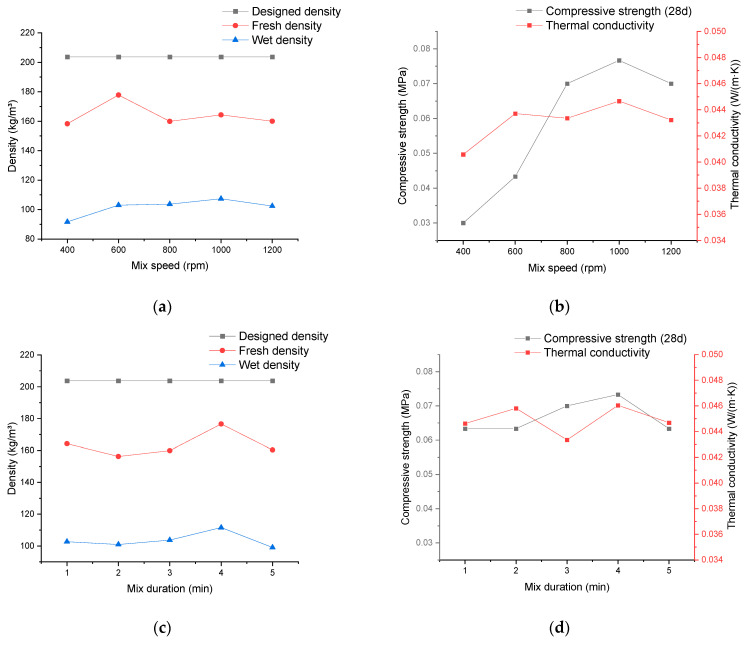
The experimental results reveal the impact of the cement paste production process on the properties of mineralized foam. (**a**) The relationship between density and mixing speed; (**b**) the relationship between thermal conductivity/compressive strength and mixing speed; (**c**) the relationship between density and mixing duration; (**d**) the relationship between thermal conductivity/compressive strength and mixing duration.

The production process of cement pastes, apart from changing equipment, can be relatively straightforwardly managed by controlling the instrument’s stirring speed and the duration of material stirring within the instrument. As depicted in Figure 5, within the parameter range given in this study, the impact resulting from altering the parameters of the mixer during the production of cement paste on various performance aspects is nearly negligible. Concerning density, as shown in Figure 5a,c, since the used material composition is the same, the designed density of the material also remains constant. Moreover, changes in speed and duration have little to no effect on the fresh density and the wet density. Regardless of the independent variable or its variations, the fresh density consistently remains within the range of 160–180 kg/m^3^, while the wet density stays in the range of 90–120 kg/m^3^. 

When considering the material’s compressive strength and thermal conductivity, as shown in Figure 5d, merely altering the stirring duration has little effect. Within the chosen stirring duration, there’s a difference of 6.23% between the maximum and minimum values of thermal conductivity and 15.79% for compressive strength. These values fluctuate without a clear trend, suggesting fluctuations that could be attributed to potential external factors during the production process, which might be considered within an acceptable range of tolerance and therefore disregarded.

However, by adjusting the mixer’s stirring speed, although it still has a limited impact on the thermal conductivity, as shown in Figure 5b, a significant upward trend is observed concerning the compressive strength. The difference between the maximum and minimum values of compressive strength reaches 155.56%. 

One possible reason for this is that by increasing the mixer’s rotational speed, the energy to which the cement particles are subjected during mixing is increased, which results in a more uniform breaking up of the otherwise agglomerated particles and leads to a more complete hydration reaction, which ultimately leads to an increase in the strength of the foam concrete without altering the raw materials used or the composition of the material. This assumption can also be supported by Figure 5b, which shows that once the mixing speed is increased to 800 rpm, the change in the compressive strength of the material is no longer evident between the mixing speed range of 800 and 1200 rpm and even falls back when the mixing speed is increased from 1000 to 1200 rpm. This represents the fact that by increasing the energy supplied by the mixer, it is indeed possible to make the hydration reaction of the cement paste more complete, but this approach reaches its limit when the mixing speed of the instrument used is increased to about 1000 rpm.

### 3.6. Relationship between Material Properties and Mixing Technologies for the Production of Mineralized Foam

**Figure 6:**
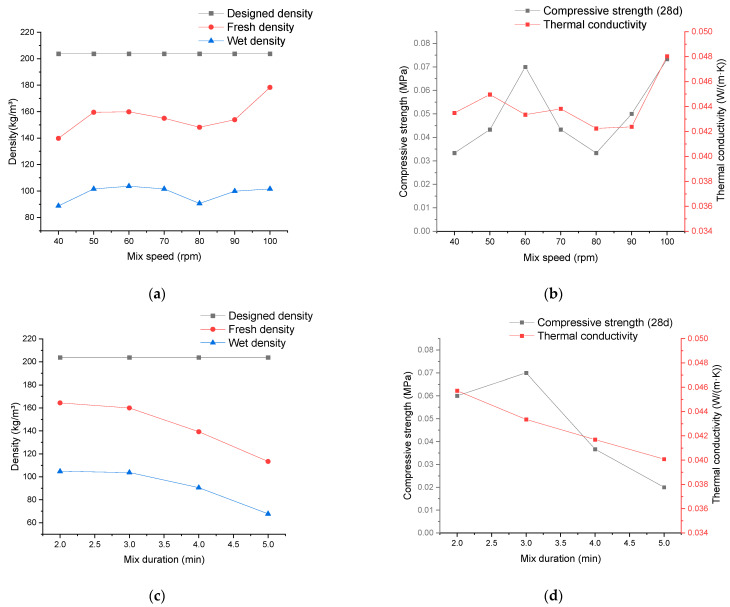
The experimental results reveal the impact of the mineralized foam production process on the properties (**a**) The relationship between density and mixing speed; (**b**) the relationship between thermal conductivity/compressive strength and mixing speed; (**c**) the relationship between density and mixing duration; (**d**) the relationship between thermal conductivity/compressive strength and mixing duration.

After the cement paste’s completion, adjustments in the mixing process of the cement paste and foam can also be made by varying the speed and duration of stirring in the ELBA mixer. The resulting impact on the properties of mineralized foam is depicted in Figure 6. Notably, the influence generated by altering the material’s stirring duration in the ELBA mixer demonstrates a distinct trend. Prolonged stirring durations lead to a decrease in both the fresh density and wet density, with a particularly pronounced effect on the fresh density, reaching a difference of up to 48.58% between the maximum and minimum values. In contrast, the variation in mixing speed within the given range shows less notable effects on material density, with the range of densities overlapping with the outcomes obtained by adjusting parameters using the CIM-30-E mixer in cement paste production.

The impact of the ELBA mixer on the compressive strength and thermal conductivity of mineralized foam is visible in Figure 6. The effect of stirring duration on the thermal conductivity of the material is evident, showing a tendency toward decreased performance with extended time. Concerning the material’s compressive strength, within the selected time range, a variation of up to 90.91% between the maximum and minimum values indicates an influence, though whether a distinct trend exists remains inconclusive. Both thermal conductivity and compressive strength demonstrate fluctuations with changes in mixing speed, with the compressive strength particularly exhibiting notable fluctuations, reaching a difference of 120% between peaks and valleys. However, in the experimental results presented in [43], there was a significant increase in material compressive strength when the mixing speed was increased from 1200 rpm to 3000 rpm. The principle behind this is mainly that the increase in mixing speed leads to a decrease in the average foam diameter [44] and a reduction in pore size in the cementitious matrix, contributing to the mechanical strength enhancement of foamed concrete [45]. Perhaps the fluctuating trend observed in this study is due to the relatively small range of variation in the given mixing speed.

### 3.7. The Microscopic Observation Results of Mineralized Foam Material

**Figure 7:**
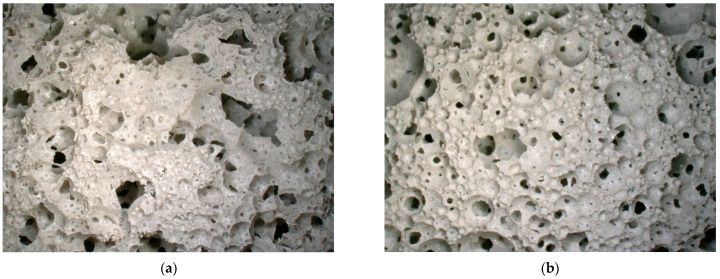
Cross-section of the sample under a microscope. (**a**) Water/cement ratio = 0.4, 20× magnification; (**b**) water/cement ratio = 1.25, 20× magnification.

Except for the samples used for compressive strength testing that were damaged, all other samples are observed under a microscope to examine the pore structure on the cross-sections. In Figure 7, two representative types of pore structures are illustrated, where Figure 7a represents a sample with a water-to-cement ratio of 0.4 and Figure 7b represents a sample with a ratio of 1.25. 

If the pore structures are roughly categorized of the material into two types, one appearing as approximately theoretically circular pores and the other as irregular voids. It can be observed that the structure represented by Figure 7a has a significant number of irregular voids alongside regularly shaped circular pore structures. In contrast, the structure represented by Figure 7b has a few irregular-shaped voids. The majority of the pores have a circular shape, and there are occasional large spherical pore structures formed by interconnected smaller ones; similar images can also be found in [12].

When comparing the parts of the structures in both images that have a similar regular shape, it can be observed that the former has a smaller average pore diameter, thinner pore walls, and complete surfaces of the pores. The latter, on the other hand, has a larger pore diameter and thicker pore walls, and the majority of the pore walls show circular perforations.

As the water-to-cement ratio increases, the presence of voids within the material gradually diminishes. Meanwhile, the likelihood of surface perforations on the pore walls increases, the thickness of the pore walls increases, and there is a widening gap in pore diameters. The most distinct transition occurs between the experimental groups with water-to-cement ratios of 0.55 and 0.60. When the ratio is at or below 0.55, voids are consistently observed in the samples, and there are occasional perforations on the pore walls. However, when the ratio exceeds 0.60, irregular voids in the samples suddenly become less evident, and a substantial number of perforations on the pore walls appear. This change does not exhibit a linear relationship with the water-to-cement ratio but rather seems to signify a sudden discrepancy.

In connection with the previous inference that the rheological properties of the cement paste affect the interaction of gas from the environment with the gas inside the material during the material fabrication process, as well as the slowing down of the decreasing trend of the viscosity/yield strength of the cement paste at a water–cement ratio of about 0.6 (see Figure 1), the following inference is proposed: Due to the fact that the rheological properties of the cement paste affect the air from the environment that enters and is retained inside the material during the fabrication process, which has no stable structure itself compared to the foam fabricated by using a foaming machine, this part of the air eventually forms an irregular cavity after being retained inside the material. The rheological properties of the cement paste also affect the stability of the pore walls, which remain intact at higher viscosities/yield strengths without perforating the surface of the final foam concrete material.

With the increase in foam content or decrease in foam density, observable changes occur in the bubble structure under the microscope. Specifically, when the foam content exceeds 20% or the foam density drops below 50 kg/m^3^, a noticeable increase in large bubble structures formed by several small bubbles connected together can be observed. However, such large pores still leave a circular-like structure rather than an irregularly shaped cavity, as shown in Figure 7a. By varying the mixing duration during the production of cement slurry, there was no noticeable change observed in the pore structure of the corresponding samples under the microscope; all remained similar to the condition depicted in Figure 7b. Similarly, during the process of making cement slurry, when the machine’s speed increased, the structure of the corresponding samples also resembled the situation shown in Figure 7b. Additionally, it was observed that the diameter of the pores tended to decrease with the increase in speed. In other words, the probability of the occurrence of large pores, notably larger than most pores as seen in the upper-left position of Figure 7b, decreased as the speed increased.

When changing the mixing duration of cement slurry and foam in the ELBA mixer, it can be observed that as the mixing time increases, the corresponding sample’s pore structure tends toward a “deterioration” state. Beyond 4 min of mixing, irregular-shaped voids similar to those in Figure 7a begin to appear on the sample surface, while samples with mixing times less than 4 min still exhibit pore structures maintaining a circular shape.

The effect of the ELBA mixer’s mixing speed on the sample structure is characterized by fluctuations, similar to the compressive strength and thermal conductivity performance of the same set of samples. With the variation in mixing speed, the observed cross-sections of the samples show an alternation between cross-sectional images where the diameter of individual pores appears nearly uniform across the entire section and images where there is a significant difference in the diameter of individual pores on the same cross-section.

Additionally, perforations appear on the pore walls of all mentioned materials, and except for the two groups of samples where the mixing time of cement slurry and foam exceeded 4 min, the foam structures in all the aforementioned cases still maintain a basic circle form.

## 4. Conclusions

In the experiment, five factors were primarily considered to evaluate their impact on the properties of mineralized foam materials: water–cement ratio, foam density, foam content, cement paste production process, and the process of mixing cement slurry with foam. This was further correlated with the rheological properties of cement paste at different water–cement ratios, as well as the difference between the designed density and the actual measured density of the materials. Based on the test results, the following conclusions were drawn:

- As the water/cement ratio increases, both the viscosity and yield strength of the cement paste decrease exponentially, with critical inflection points at approximately 0.6 water/cement ratio. Simultaneously, the difference between the designed and fresh densities reduces with the increasing ratio, exhibiting a slower reduction trend between 0.6 and 0.8 in the water/cement ratio. However, the variation in wet density remains relatively small. Considering the ELBA mixer used for blending the cement paste and foam, it continuously transfers the material from the bottom of the drum to the top, allowing it to fall back down, inevitably introducing a significant amount of gas from the environment into the mixture. The higher viscosity and yield strength of the cement paste post-mixing retain more air, which can explain the density variation in the samples. This phenomenon also explains the presence of numerous irregular-shaped voids in samples with a water/cement ratio below 0.6, while those above this value exhibit almost no voids. Higher ratios result in excessively low viscosity and yield strength of the cement paste, preventing stable foam structure maintenance and causing perforations on the surface of pore structures. This elucidates why the material’s strength peaks at a water–cement ratio of 0.75 before declining and why the material’s thermal conductivity increases with the water–cement ratio until approximately 0.6 and fluctuates afterward within a certain range.
- As the foam density increases, there is a significant enhancement observed in the material’s overall density, thermal conductivity, and compressive strength. Conversely, an increase in foam content results in a decline in material density, compressive strength, and thermal conductivity. Particularly notable is the noticeable impact of foam content on the material’s compressive strength. As the water–cement ratio used for sample production remains constant at 0.6, the difference between the designed and measured densities consistently maintains relative stability.
- In the cement paste production process, only the mixing speed moderately affects the compressive strength of mineralized foam. Higher speeds can enhance the sample’s strength without influencing other properties. Conversely, the time consumed in cement paste production has a minimal impact on the final material properties.
- The blending process of cement paste and foam is significantly influenced by machinery and procedures. Specifically, the mixing duration exhibits a trend where prolonged stirring results in a higher probability and volume of environmental air entering the material. This leads to a decline in both fresh and wet densities, with a more pronounced impact on the fresh density. With increased air content, a reduction in the thermal conductivity and strength of the material is observed. However, the impact of the selected mixing speed in the range of this study during mixing remains less conclusive. It’s possible that at speeds below 60 rotations per minute, higher speeds contribute to a more uniform material mixture. On the other hand, higher speeds might disrupt the original foam structure, allowing a significant influx of environmental air into the material, followed by its release, resulting in unpredictable consequences.

## 5. Outlook

In this study, to ensure clarity in the relationship between variables and provide directions for future improvements, material compositions and techniques were simplified as much as possible. For example, the rheological properties of the cement slurry can be adjusted using superplasticizers and viscosity enhancement admixtures. Improvements in material structure can be made by using fillers such as fly ash. Therefore, there is considerable potential evident in the current results. In future studies, efforts will focus on improving the pore structure of the material and altering material compositions to further reduce thermal conductivity without significantly affecting material strength. Alternatively, efforts may be directed towards enhancing the strength of materials with low thermal conductivity while further simplifying and standardizing the sample preparation process.

## Figures and Tables

**Table 1:** Material composition of cement.

Material Composition	Content (wt%)
Portland cement clinker	65–79
Calcined schale	6–29
Limestone	6–19

**Table 2:** Test parameters.

Parameters	Variables for Test	Mixture Type
water/cement Ratio (%)	40, 45, 50, 55, 60, 65, 70, 75, 80, 85, 90, 95, 100, 105, 110, 115, 120, 125, 130	Paste
Foam density (kg/m^3^)	30, 50, 70, 90	Concrete
Foam content (%)	10, 15, 20, 25, 30, 35, 40	Concrete
CIM 30 E Mixing speed (rpm)	400, 600, 800, 1000, 1200	Concrete
CIM 30 E Mixing duration (min)	1, 2, 3, 4, 5	Concrete
ELBA Mixing speed (rpm)	40, 50, 60, 70, 80, 90, 100	Concrete
ELBA Mixing duration (min)	2, 3, 4, 5	Concrete

**Table 3:** Mix proportions.

Parameters	Name of Specimens	W/C-Ratio (%)	Foam Density (kg/m^3^)	Unit Content (kg/m^3^)			CIM-30E		ELBA	
				Water	Cement	Foam	rpm	min	rpm	min
W/C-ratio	MS-WZ	40	45	47.03	117.57	41.15	800	3	60	3
	MS-WZ	45	45	50.94	113.21	41.04	800	3	60	3
	MS-WZ	50	45	54.58	109.16	40.93	800	3	60	3
	MS-WZ	55	45	57.96	105.39	40.84	800	3	60	3
	MS-WZ	60	45	61.12	101.87	40.75	800	3	60	3
	MS-WZ	65	45	64.07	98.58	40.66	800	3	60	3
	MS-WZ	70	45	66.84	95.49	40.58	800	3	60	3
	MS-WZ	75	45	69.44	92.59	40.51	800	3	60	3
	MS-WZ	80	45	71.89	89.86	40.44	800	3	60	3
	MS-WZ	85	45	74.20	87.29	40.37	800	3	60	3
	MS-WZ	90	45	76.38	84.86	40.31	800	3	60	3
	MS-WZ	95	45	78.44	82.57	40.25	800	3	60	3
	MS-WZ	100	45	80.39	80.39	40.20	800	3	60	3
	MS-WZ	105	45	82.24	78.33	40.14	800	3	60	3
	MS-WZ	110	45	84.00	76.37	40.09	800	3	60	3
	MS-WZ	115	45	85.68	74.50	40.05	800	3	60	3
	MS-WZ	120	45	87.27	72.73	40.00	800	3	60	3
	MS-WZ	125	45	88.79	71.03	39.96	800	3	60	3
	MS-WZ	130	45	90.24	69.42	39.91	800	3	60	3
Foam density	MS-L5W70	60	30	42.07	70.12	28.05	800	3	60	3
	MS-L5W90	60	50	67.21	112.01	44.80	800	3	60	3
	MS-L5W110	60	70	90.33	150.55	60.22	800	3	60	3
	MS-L3W120	60	90	111.68	186.14	74.46	800	3	60	3
Foam content	MS-SG10	60	45	133.83	223.04	35.69	800	3	60	3
	MS-SG15	60	45	95.83	159.71	38.33	800	3	60	3
	MS-SG20	60	45	74.64	124.39	39.81	800	3	60	3
	MS-SG25	60	45	61.12	101.87	40.75	800	3	60	3
	MS-SG30	60	45	51.75	86.25	41.40	800	3	60	3
	MS-SG35	60	45	44.87	74.78	41.88	800	3	60	3
	MS-SG40	60	45	39.60	66.01	42.24	800	3	60	3
CIM 30 E Mixing speed	MS-GDZ400	60	30	61.12	101.87	40.75	400	3	60	3
	MS-GDZ600	60	50	61.12	101.87	40.75	600	3	60	3
	MS-GDZ800	60	70	61.12	101.87	40.75	800	3	60	3
	MS-GDZ1000	60	90	61.12	101.87	40.75	1000	3	60	3
	MS-GDZ1200	60	45	61.12	101.87	40.75	1200	3	60	3
CIM 30 E Mixing duration	MS-GMZ1	60	45	61.12	101.87	40.75	800	1	60	3
	MS-GMZ2	60	45	61.12	101.87	40.75	800	2	60	3
	MS-GMZ3	60	45	61.12	101.87	40.75	800	3	60	3
	MS-GMZ4	60	45	61.12	101.87	40.75	800	4	60	3
	MS-GMZ5	60	45	61.12	101.87	40.75	800	5	60	3
ELBA Mixing speed	MS-ADZ40	60	45	61.12	101.87	40.75	800	3	40	3
	MS-ADZ50	60	45	61.12	101.87	40.75	800	3	50	3
	MS-ADZ60	60	45	61.12	101.87	40.75	800	3	60	3
	MS-ADZ70	60	45	61.12	101.87	40.75	800	3	70	3
	MS-ADZ80	60	45	61.12	101.87	40.75	800	3	80	3
	MS-ADZ90	60	45	61.12	101.87	40.75	800	3	90	3
	MS-ADZ100	60	45	61.12	101.87	40.75	800	3	100	3
ELBA Mixing duration	MS-AMZ2	60	45	61.12	101.87	40.75	800	3	60	2
	MS-AMZ3	60	45	61.12	101.87	40.75	800	3	60	3
	MS-AMZ4	60	45	61.12	101.87	40.75	800	3	60	4
	MS-AMZ5	60	45	61.12	101.87	40.75	800	3	60	5

## Data Availability

The data supporting the conclusions of this article are available by the authors upon request. The data are not publicly available due to [This project is not yet fully completed, all experimental data will be released upon completion of the project].
